# Emissions of nitrous oxide and ammonia from a sandy soil following surface application and incorporation of cauliflower leaf residues

**DOI:** 10.1017/S0021859615000027

**Published:** 2015-02-25

**Authors:** L. NETT, R. FUß, H. FLESSA, M. FINK

**Affiliations:** 1 Leibniz-Institute of Vegetable and Ornamental Crops Großbeeren and Erfurt, Theodor-Echtermeyer-Weg 1, 14979, Großbeeren, Germany; 2 Johann Heinrich von Thünen Institute, Institute of Climate-Smart Agriculture, Bundesallee 50, 38116 Braunschweig, Germany

## Abstract

Vegetable production systems are often characterized by excessive nitrogen (N) fertilization and the incorporation of large amounts of post-harvest crop residues. This makes them particularly prone to ammonia (NH_3_) and nitrous oxide (N_2_O) emissions. Yet, urgently needed management strategies that can reduce these harmful emissions are missing, because underlying processes are not fully understood. The present study therefore focuses on the effects of residue placement on NH_3_ and N_2_O emissions. For this, cauliflower leaf residues (286 kg N/ha) were either applied as surface mulch (mulch) or mixed with the topsoil (mix) and *in situ* NH_3_ and N_2_O emissions were investigated. The experiment took place on a sandy soil in Northeastern Germany during summer 2012. Residue application created a high peak in N_2_O emissions during the first 2 weeks, irrespective of residue placement. There was no significant difference in the emission sums over the experimental period (65 days) between the mix (5·8 ± 0·68 kg N_2_O-N/ha) and the mulch (9·7 ± 1·53 kg N_2_O-N/ha) treatment. This was also the case for NH_3_ emissions, which exhibited a lower initial peak followed by a prolonged decline. Measured emission sums were 4·1 ± 0·33 (mix) and 5·1 ± 0·73 (mulch) kg NH_3_-N/ha. It was concluded that substantial NH_3_ and N_2_O emissions can occur after high input of available organic carbon and N even in a coarse-textured soil with low water-holding capacity. Other than expected, surface-application does not enhance NH_3_ emissions at the expense of N_2_O emissions compared with residue mixing into the soil, at least under the conditions of the present study.

## INTRODUCTION

The application of synthetic nitrogen (N) fertilizers has boosted crop yields and hence sustained the rapidly growing world population in recent decades (Galloway *et al.* 2004). As a downside, the translocation of reactive N compounds from agricultural to other environmental systems has increased, e.g. in the form of gaseous N emissions. These emissions have negative environmental consequences, such as the contribution of nitrous oxide (N_2_O) to global warming (Forster *et al.* 2007) and the depletion of stratospheric ozone (Ravishankara *et al.* 2009) as well as the acidification and eutrophication of ecosystems caused by ammonia (NH_3_) deposition (Kuylenstierna *et al.* 1998; Robertson & Vitousek 2009). Therefore, strategies to reduce gaseous N losses from agricultural and horticultural systems are urgently needed.

The production of many field vegetable crops is particularly prone to gaseous N emissions due to the high input of readily available carbon (C) and N in crop residues after harvest. For instance, cauliflower, broccoli and Brussels sprouts typically contain more than 150 kg N/ha in crop residues, according to Feller *et al.* (2010). These residues are usually chopped (e.g. by a flail mower) and subsequently incorporated into the soil. Depending on the management strategy and environmental conditions, incorporation can either be conducted immediately after chopping or with a time-lag of up to a few weeks. The magnitude of NH_3_ losses from surface-applied vegetable residues has been reported to range between 50 and 160 mg NH_3_-N/g residue-N in 4 months depending on residue C:N ratio and total N content (de Ruijter *et al.* 2010*a*). Moreover, NH_3_ losses in many cases appear to be a surface phenomenon that almost disappears after incorporation of the source substrate into the soil (Glasener & Palm 1995; Mohr *et al.* 1998; de Ruijter *et al.* 2010*a*, *b*). Owing to the complex mineralization–immobilization turnover and the variability in the abiotic conditions that determine NH_3_ creation and volatilization (e.g. soil diffusivity, air turbulence, pH value), NH_3_ losses are still difficult to predict (Ni 1999).

Unlike NH_3_ emissions, non-ammoniacal nitrogenous gases can be derived from a number of different biotic soil processes. According to Davidson *et al.* (2000), the total losses of nitric oxide (NO), N_2_O and nitrogen gas (N_2_) via nitrification and denitrification are controlled by an ecosystem's N cycling rate, while the relative contributions of these compounds are mostly determined by abiotic factors, predominantly oxygen (O_2_) availability. However, during the last decade, a series of further microbial processes involving partly or complete nitrification and/or denitrification has been described (Baggs 2008). These processes can be associated with specific ecological niches, e.g. the combination of C, N and O_2_ availabilities that govern their occurrence (Wrage *et al.* 2001). These availabilities are very difficult to predict exactly, in particular when recently incorporated pieces of readily decomposable plant residues create micro-environments of high microbial activity, nutrient turnover and O_2_ consumption (Flessa & Beese 1995; Azam *et al.* 2002).

Thus, the high spatial and temporal variability in the biotic and abiotic factors controlling the source processes of N_2_O complicate the prediction of net emissions. The Intergovernmental Panel on Climate Change (IPCC) uses a default emission factor for N_2_O with respect to any kind of N input into mineral arable soil of 10 mg N_2_O-N/g N, i.e. the proportion of applied N lost as N_2_O in a year (IPCC 2006). This may also be a good approximation for an average emission factor with respect to N_2_O derived from crop residues according to a meta-analysis by Novoa & Tejeda (2006). However, for vegetable crop residues with a high water content and low C:N ratio, N_2_O loss may exceed the emission factor's upper limit of 30 mg N_2_O-N/g N given by the IPCC (2006), as reported by Velthof *et al.* (2002) and Ruser *et al.* (2009).

Most studies on nitrogenous emissions from crop residues do not represent natural field conditions. They have been conducted under laboratory or greenhouse conditions, using vessels or containers that create unnatural physical soil conditions. Often plant residues were dried and cut into much smaller pieces than common in agricultural practice and experiments were performed at constant temperature and moisture conditions. Therefore, research results are often not relevant to practical implementation. Chen *et al.* (2013) performed a meta-analysis on data from studies investigating N_2_O emissions after crop residue application and clearly demonstrated that laboratory incubation conditions produce emission factors that are generally higher than those found in field studies. However, the number of existing field studies is limited, in particular those considering the simultaneous emissions of NH_3_ and N_2_O. Such studies are crucial to assess the integrated environmental impact of a particular treatment, since fluxes of these gases can be negatively correlated. The effects of management practices on the combined emissions of NH_3_ and N_2_O in vegetable production systems are poorly understood but could have the potential to mitigate N emissions from these systems.

The objectives of the present study were to determine the effects of cauliflower residue placement (surface-application *v.* incorporation) on C mineralization dynamics and the emissions of N_2_O and NH_3_. It was hypothesized that NH_3_ emissions would be higher after surface-application than after incorporation of residues and vice versa for N_2_O emissions. The study was carried out on a sandy soil under semi-controlled field conditions (irrigation and shading).

## MATERIALS AND METHODS

### Experimental site

The experiment was performed at Großbeeren (52°21′N, 13°18′E, 42 m a.s.l.), 20 km south of Berlin, Germany. The soil type was an Arenic Luvisol, with a pH value of 5·3 (calcium chloride (CaCl_2_)) and a fine sand texture with 910, 40 and 50 g/kg sand, silt and clay, respectively. This coarse-textured soil could be considered susceptible to N leaching losses due to a low water-holding capacity. However, as a result of several practical advantages associated with a coarse soil texture, such as facilitated heating-up in spring as well as better trafficability and workability, vegetable production is common on such soils in Germany. The average annual precipitation amounted to 500 mm/yr. Mean annual temperature constituted 9·8 °C with an average of 38 frost-days per year. Total soil C stocks were 11·6 and 11·8 t C/ha, total soil N stocks were 0·85 and 0·88 t N/ha, bulk densities were 1·34 and 1·42 t/m^3^, and total porosities were 0·48 and 0·44 m^3^/m^3^ in the soil depth intervals 0–0·1 and 0·1–0·2 m, respectively. The experimental plot had been cropped with barley or wheat for at least 10 years until 2010. Cereals received *c*. 70 kg N/ha annually and were harvested completely leaving only the stubble for incorporation with the disc harrow. The last cereal harvest on the experimental plot took place in autumn 2010, followed by a fallow period until the start of the experiment, which was maintained by herbicide application and soil tillage.

### Gas flux measurements

#### Chambers

The static closed chamber technique was applied using two-part chambers made from polyvinyl chloride (PVC) with a circular base area of 0·1152 m^2^ and a volume of 40·3–46·3 litres (depending on the actual height after incorporation). The chamber design principally followed the recommendations by Parkin & Venterea (2010). Chambers were equipped with a vent (15 mm diameter, 0·4 m length), reflective foil and a gasket o-ring seal in combination with fasteners to provide a gas-tight connection between chamber anchor and chamber top. The chamber anchors (0·3 m height) were inserted to a depth of 0·2 m, so that a collar of 0·1 m remained above the soil surface. Fans (diameter 38 mm, wind speed 1–2 m/s at 0·15 m distance) were attached to the inner wall of the anchors, directed parallel to the soil surface alongside the anchor wall. These fans ran continuously from 11 July onwards. The anchor collar height and ventilation deviated from the protocol of Parkin & Venterea (2010) and were necessary to guarantee that air turbulence within the chambers was not limiting NH_3_ emissions, especially during chamber closure.

#### Nitrous oxide and carbon dioxide samples

Gas samples of 30 ml were taken through a septum-port using a gas-tight syringe and filled into pre-evacuated 20 ml glass vials sealed with butyl rubber stoppers at 0, 20, 40 and 60 min after chamber closure. Gas samples were analysed for concentrations of carbon dioxide (CO_2_) and N_2_O using a gas chromatograph (GC-2014, Shimadzu Europa GmbH, Duisburg, Germany), modified according to Loftfield *et al*. (1997). Briefly, a 1 m Porapak Q pre-column (to remove water with a back-flush) and a 3 m main column at constant temperature of 65 °C were coupled to a ^63^Ni electron capture detector operating at 320 °C and N_2_ was used as carrier and make-up gas. Sampling was performed with an auto-sampler and by connecting the evacuated 1 ml sampling loop to the sample vial followed by automated equilibration to ambient pressure. The concentrations derived from the chromatograph were corrected for the dilution caused by the residual air left in the vials after pre-evacuation for sampling. The slope of concentration *v*. time was calculated using a robust regression with a Huber-M estimator (Huber 1981). Then, gas fluxes were calculated considering the ambient air temperature and pressure during measurement as well as the individual chamber height.

#### Ammonia samples

Cellulose filters (90 mm diameter, type 15A, Carl Roth GmbH, Karlsruhe, Germany) were washed in deionized water (2 × 30 min ultra-sound bath), dried at 60 °C, impregnated (2 ml/filter) with phosphoric acid solution (33 g H_3_PO_4_/l methanol:H_2_O solution (9:1)), dried in pure N_2_, and stored in sets of three in centrifuge tubes, sealed with lids and plastic paraffin film. For measurement, the filter sets were unpacked, immediately clamped on specifically designed plastic rings, which were fitted on the inner wall of the chamber tops, and the chamber was closed for 1 h. After exposure in the ventilated chambers, filters were collected and put in centrifuge tubes, sealed with lids and plastic paraffin film, and stored at −18 °C until analysis. For analysis, three filters were extracted with 40 ml of deionized water for 1 h in an ultra-sound bath and the extract analysed for ammonium (NH_4_^+^-N) concentration using an Eppendorf Patient Oriented System (EPOS) analyser (Eppendorf, Hamburg, Germany). Assuming that NH_3_ emitted during chamber closure was completely trapped by the filters, the NH_3_-N emission rate was calculated as the amount of extracted NH_4_^+^-N divided by the chamber closing time. On all measurement dates, unexposed filters were analysed in the same way and values subtracted from all exposed treatments. A pre-experiment was performed to test the uptake capacity of the filters and the recovery rate of the extraction procedure. For this, different amounts of NH_3_ calibration gas (20 ml NH_3_/l N_2_) were injected into a desiccator (22·43 litres), which was equipped with a fan for ventilation (diameter 24 mm, wind speed 0·1–0·3 m/s at 0·15 m distance) and contained three filters prepared as described above. After an exposure time of 1 h, the average (± s.e.m.; *n* = 3) recoveries of the injected amounts (0·29, 0·77 and 2·30 mg NH_3_-N) were 108 ± 1·2, 105 ± 2·3 and 105 ± 2·1%, respectively. Hence, apart from the slight overestimation that was observed, the pre-experiment indicated that this method was suitable for trapping high amounts of NH_3_ in the short time frame of 1 h and that the quantitative extraction worked well.

### Cumulative emissions

Cumulative emissions were obtained by converting the observed hourly emission rates of CO_2_-C, N_2_O-N and NH_3_-N into daily emission rates and interpolating linearly between the measurement dates. To enable the comparison of temporal dynamics in gas emissions, the experimental period was divided into two phases: an early period of clearly enhanced emission rates in the amendment treatments (1–15 days after residue application (DAA)) and a late period containing the rest of the experimental period (16–66 DAA). Cumulative emissions were related to the total residue C and N input to show the significance of these fluxes. For this, the cumulative emissions of the control treatment were subtracted from those of the considered treatment and the result was divided by the amount of C (or N) applied through cauliflower residues. It should be noted that these emission factors for residues (EFRs) do not imply that the emitted CO_2_-C, N_2_O-N and NH_3_-N originated exclusively from the residue C and N pools, since priming effects may have changed the transformation processes of soil C and N. Consequently, EFRs should be interpreted as reflecting the emissions induced by the residue application.

### Experimental design

On 7 June 2012, soil was tilled to a depth of 0·2 m using a rotary spading machine. Between 13 and 15 June, chamber anchors were driven into the soil. On 22 June, soil moisture (time domain reflectometry, TDR) and temperature sensors were inserted into the soil (probe rods parallel to the soil surface) between the chamber anchors at nine locations and at soil depths of 0·05 and 0·15 m. Also, a cup anemometer was put in place at 0·4 m above the soil surface between the chamber anchors. On 10 July two identical roofs of 5 × 5 m dimensions (open to the sides, height 1·5–2·0 m) were installed, which served as a protection against precipitation and high solar irradiation by using a combination of transparent foil and shading screens. The main intention of this roofing was to avoid peak N_2_O emissions induced by extreme rain events and to enable the control of soil moisture by irrigation, which was executed regularly and homogeneously using a spray lance. These artificial conditions may have altered the absolute magnitude of gas emissions but facilitated the comparison of treatments, which was the key target of the present study.

The first gas flux measurement took place on 13 July 2012, prior to any fertilizer or residue application. Immediately after measurements were completed, all chamber anchors received an inorganic N fertilization (930 mg NO_3_-N+70 mg NH_4_^+^-N per g N), dissolved in 200 ml of distilled water, corresponding to an equivalent amount of 30 kg N/ha. Thereby, the total inorganic N content of the soil was raised to 41 kg N/ha in 0–0·2 m. This corresponded to the expected post-harvest soil inorganic N content when cauliflower is fertilized according to Good Agricultural Practice (300–350 kg N/ha; Feller *et al.* 2010). On 16 July, another pre-measurement of gas fluxes was performed followed by the application of slashed cauliflower leaves in the respective treatments. The slashing of cauliflower leaves was performed using a cutting machine without blades, which produced residues that were similar to those left by a flail mower. Three treatments, an unamended control (co), surface-applied cauliflower leaves (mulch) and cauliflower leaves mixed homogeneously with the soil layer 0–0·15 m (mix) were established within the chamber anchors. The cauliflower leaf residues (365 g C and 31 g N per kg dry mass (60 °C; CNS-Analyser VARIO EL, Elementar, Hanau, Germany) were applied at a rate equivalent to 3367 kg C and 286 kg N/ha in both amended treatments. This rate could be considered exceptionally high as compared with values for conventionally harvested cauliflower (153–180 kg N/ha; Feller *et al.* 2010). However, such high amounts commonly occur in practice when the complete or a high proportion of the crop is incorporated into the soil due to insufficient crop quality. Hence, the present experiment represented a worst-case scenario with respect to the amount of cauliflower leaf residues.

Separate chambers were used for NH_3_ and N_2_O/CO_2_ flux measurements to avoid potential effects of NH_3_ filter traps on N_2_O or CO_2_ as well as to allow independent chamber closure times. Using three replicate chambers per treatment, this gave a total of 18 chamber anchors, nine under each roof with treatments arranged in Latin square designs. Gas flux measurements were done on 1, 4, 9, 14, 21, 28, 37, 44 and 65 DAA.

### Statistics

Statistical analyses were performed using the R statistics software (v. 3·0·0; R Development Core Team 2013). For parametric tests, the assumption of normal distribution of within-group errors was tested by the Kolmogorov–Smirnov test, while homoscedasticity was checked using Levene's test. When analysis of variance yielded a significant effect of the factor ‘residue placement’, comparisons among the three amendment treatments were performed using Tukey's HSD test. Also, comparisons between the control and the combined mean of the amended treatments were done using linear contrasts (control +1·0, mix −0·5, mulch −0·5). Emission factors for residues were compared using Welch's two-sample t test. Unless stated otherwise, results are presented as mean ±1 standard error of the mean (S.E.M.). Statistical significance was stated at *P* < 0·05.

## RESULTS

### Abiotic conditions

**Fig. 1.**
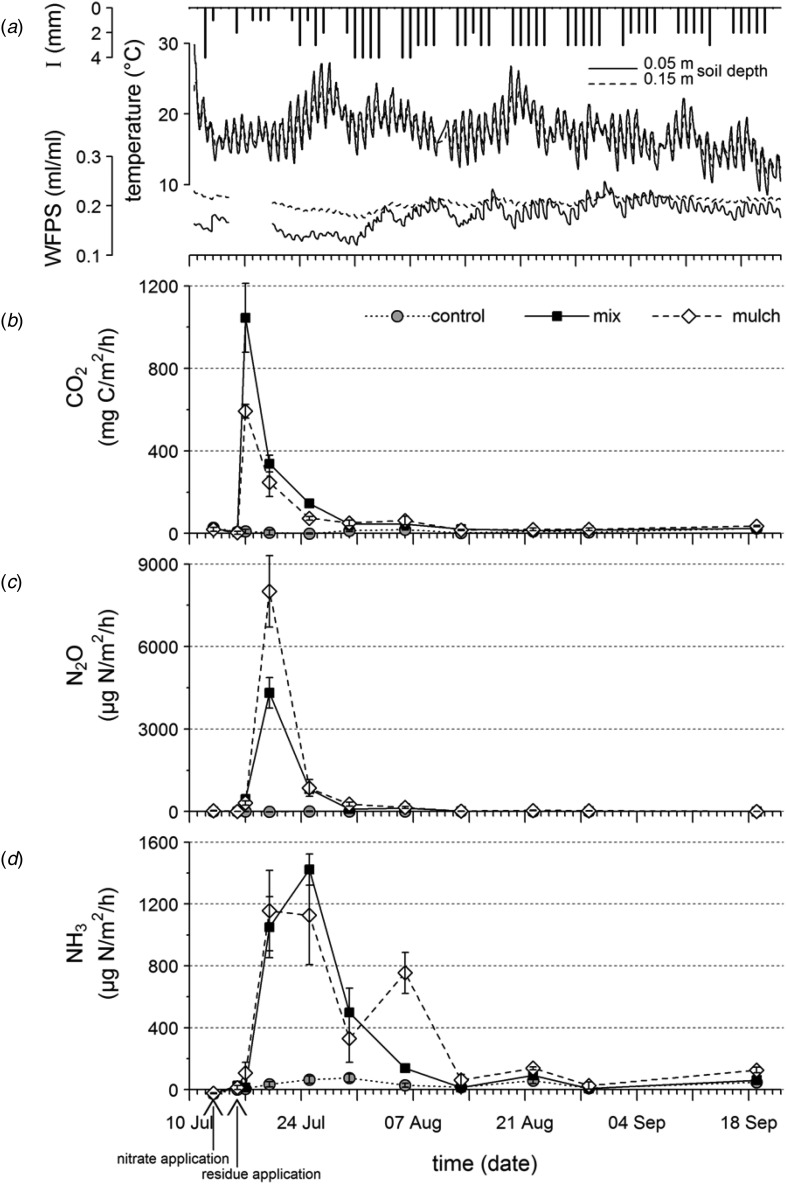
Time course during the experiment of (*a*) abiotic conditions, where I=irrigation in mm, WFPS = water-filled pore space in ml/ml at 0·05 and 0·15 m soil depth (*n* = 9), and temperature in °C at 0·05 and 0·15 m soil depth (*n* = 9); (*b*) CO_2_ emission rates in mg C/m^2^/h; (*c*) N_2_O emission rates in μg N/m^2^/h, and (*d*) NH_3_ emission rates in μg N/m^2^/h for the treatments with surface application (mulch) and incorporation (mix) of cauliflower residues and the control without residues. Error bars indicate standard errors of the mean (*n* = 3).

The irrigation sum during the experiment was 123 mm with daily amounts of 0–4 mm, which kept the soil moisture at an average (*n* = 1525) of 0·18 (min. 0·12, max. 0·25) and 0·21 (min. 0·18, max. 0·23) ml/ml water-filled pore space (WFPS) at a soil depth of 0·05 and 0·15 m, respectively (Fig. 1*a*). These values corresponded to volumetric water contents per total soil volume of 0·088 and 0·091 ml/ml and were derived using average soil porosities according to undisturbed soil core saturation measurements. These water contents can be regarded as high and approximately reflect the soil's field capacity. The field capacity was not determined. However, water contents at time of installing the roofs (10 July 2012; Fig. 1*a*) did not exceed water contents during the experimental period, even though precipitation summed up to 75 mm within 2 weeks before installing the roofs. This suggests a field capacity of approximately 0·10 ml/ml relating to soil total volume. During the first 3 weeks of the experiment (until 1 August), soil moisture tended to be lower with 0·14 (min. 0·12, max. 0·18) ml/ml WFPS (*n* = 325) as compared to 0·19 (min. 0·14, max. 0·25) ml/ml WFPS (*n* = 1200) in the remaining period. This was a result of very high soil temperatures and lower irrigation rates (Fig. 1*a*). Note that there was a gap in soil moisture data during the first week after residue application due to a data logger failure. Towards the end of the experiment lower irrigation rates were necessary to keep soil moisture roughly constant, due to gradually decreasing temperatures (Fig. 1*a*). The median wind speed was 0·2 m/s and the relative frequency of wind speeds over time was 0·17, 0·35, 0·29 and 0·19 for the intervals 0–0·1, 0·1–0·2, 0·2–0·3 and 0·3–2·0 m/s, respectively (*n* = 1633, hourly recordings to an accuracy of ±0·1 m/s, data not shown).

### Carbon dioxide emissions

Since 1-h chamber closing times are too long for unbiased measurement of soil respiration, the emission values presented should only be interpreted as a relative proxy of soil microbial activity and dynamics of residue mineralization.

Before the application of cauliflower residues as well as throughout the experiment in the control treatment, CO_2_ emission rates were generally low and did not exceed rates of 30 mg CO_2_-C/m^2^/h (Fig. 1*b*). After the application of cauliflower residues, the emission rates of CO_2_ increased immediately with highest rates observed 1 day after application, followed by a sharp decrease and approaching the rate of the control treatment 4 weeks later (Fig. 1*b*). The cumulative CO_2_-C emissions of the three amendment treatments (Table 1) were significantly (*F*-test: *P* < 0·001) different in the early period (Tukey's HSD test: mix > mulch > control) but not in the late period (*F*-test: *P* = 0·090). This difference was also apparent in the EFRs, which indicated that 301 and 186 mg CO_2_-C/g residue-C had been respired to CO_2_ during the early period in the mix and mulch treatment, respectively (Welch Two Sample *t* test: *P* = 0·036; Table 1). According to linear contrasts, the cumulative emissions of the combined amended treatments were significantly higher than those of the control treatment in the early (*P* < 0·001) and late (*P* = 0·042) period as well as over the total duration (*P* < 0·001) of the experiment. No significant differences occurred between mix and mulch treatments in emission sums (Tukey's HSD tests) or EFRs (Welch Two Sample *t* tests), either in the late period or over the whole experimental period.

**Table 1.** Cumulative emissions of CO_2_, N_2_O and NH_3_ and emission factors for residues (EFRs) for the early, late and total experimental period. All values (means±s.e.m.) are based on three replicate chamber anchors

Period	Treatment	Cumulative emission (kg C or N per ha)			Emission factors for residues (EFR)* (mg C or N per g residue-C or -N)		
		CO_2_	N_2_O	NH_3_	CO_2_	N_2_O	NH_3_
Early (1–15 DAA†)	Control	19 ± 19·9	0·0 ± 0·00	0·2 ± 0·04	n.a.	n.a.	n.a.
	Mix	1034 ± 94·5	5·4 ± 0·60	3·1 ± 0·24	301 ± 28·1	19 ± 2·1	10 ± 0·8
	Mulch	647 ± 77·6	9·1 ± 1·49	2·8 ± 0·55	186 ± 23·0	32 ± 5·2	9 ± 1·9
Late (16–66 DAA)	Control	144 ± 21·0	0·0 ± 0·01	0·4 ± 0·05	n.a.	n.a.	n.a.
	Mix	294 ± 52·2	0·4 ± 0·10	1·0 ± 0·10	45 ± 15·5	1 ± 0·3	2 ± 0·3
	Mulch	368 ± 86·2	0·6 ± 0·07	2·3 ± 0·50	67 ± 25·6	2 ± 0·3	7 ± 1·7
Total (1–66 DAA)	Control	164 ± 35·0	0·0 ± 0·01	0·6 ± 0·06	n.a.	n.a.	n.a.
	Mix	1329 ± 145·7	5·8 ± 0·68	4·1 ± 0·33	346 ± 43·3	20 ± 2·4	12 ± 1·2
	Mulch	1016 ± 139·7	9·7 ± 1·53	5·1 ± 0·73	253 ± 41·5	34 ± 5·3	16 ± 2·5

* Cumulative emissions of respective treatment minus those of control treatment divided by amount of C or N in cauliflower leaves (3367 kg C/ha, 286 kg N/ha).

† Days after residue application.

n.a., not applicable.

### Nitrous oxide emissions

The N_2_O emission rates exhibited a high peak after cauliflower residue application (Fig. 1*c*), which was more short-lived and appeared delayed in comparison with CO_2_ emissions. The emission sums and EFRs in the early and late period, respectively, were 1·7- and 1·5-fold higher in the mulch treatment than in the mix treatment (Table 1) but these differences were not statistically significant according to Tukey's HSD tests (emissions sums) or Welch Two Sample *t* tests (EFRs). Nitrous oxide emissions were not detectable, or were negligible, before the application of cauliflower residues as well as throughout the experiment in the control treatment (Fig. 1*c*; Table 1).

### Ammonia emissions

The NH_3_ emission rates responded as quickly to residue application as the N_2_O emission rates did, but showed a prolonged decline thereafter (Fig. 1*d*). The cumulative emissions of NH_3_-N after cauliflower application were slightly lower than the N_2_O-N emissions, while the control treatment showed minor but detectable NH_3_-N emissions (Table 1). The emission sums of the amended treatments in the early and late period, respectively, were 15·5 and 2·5 times (mix) and 14 and 5·8 times (mulch) higher than those of the control treatment (Table 1). These differences were statistically significant according to Tukey's HSD tests (*P* < 0·05) with the exception of mix and control treatments in the late period. No significant differences were found in EFRs between the two amended treatments in any of the investigated time periods (Welch Two Sample *t* tests).

## DISCUSSION

### Nitrous oxide emissions

According to Davidson *et al.* (2000), N_2_O production is highest when WFPS ranges between 0·50 and 0·70 ml/ml, while the dominating source of N_2_O switches from nitrification at lower WFPS to denitrification at higher WFPS. The low values of WFPS (<0·25 ml/ml) that were observed in the present study while regular irrigation was applied reflect the low water-holding capacity of this sandy soil. In spite of this fact, the observed N_2_O losses reached a high magnitude, both in terms of emission rates and sums. This can be explained by the high input of crop residues that on the one hand featured a water content of 0·89 g H_2_O/g FM (data not shown) and on the other hand delivered readily available organic C, which increased microbial respiration and thereby probably created anaerobic microsites of increased denitrification rates. Reduced redox potentials and hence anaerobicity in the vicinity of crop residues in soil have been reported by Flessa & Beese (1995), who studied sugar beet leaf decomposition and associated N_2_O production in a microcosm experiment. In the present study, the anoxic microsites around the cauliflower leaf residues, accompanied by the high availabilities of organic C as energy source (e^−^-donor) and NO_3_^−^ as alternative e^−^-acceptor probably sustained high denitrification rates (Azam *et al.* 2002). The NO_3_^−^ was presumably derived from both pre-existing soil NO_3_^−^ and nitrification of ammonified residue-N from aerobic sites in the proximity of denitrification ‘hot spots’. Although the magnitude of N_2_O emissions observed here indicate that local anaerobicity occurred, it is possible that coupling of autotrophic nitrification and aerobic denitrification accounted for a relevant proportion of N_2_O emissions (Bateman & Baggs 2005).

The N_2_O emission sums (5·8–9·7 kg N/ha) and EFRs (20–34 mg N_2_O-N/g residue-N) obtained for a period of 65 days in the present study were high in comparison with those from other studies featuring vegetable residues. For instance, Baggs *et al.* (2000) investigated N_2_O emissions after application of grass, grass/clover, lettuce, cereal and oilseed rape residues and reported highest emissions after rotary tillage of lettuce residues (1·6 t DM/ha, C:N = 7·5), which amounted to 1·6 kg N_2_O-N/ha in 79 days. Emission factors given in the literature ranged from 2 to 17 mg N_2_O-N/g residue-N in 185–203 days for sugar beet and pea residues (Harrison *et al.* 2002), from −1 to 5 mg N_2_O-N/g residue-N in 200–243 days for sugar beet (C:N = 22–34) and soybean (C:N = 31–46) residues (Koga 2013) and from 7 to 9 mg N_2_O-N/g residue-N in 60 days for onion leaf (C:N = 12) and soybean (C:N = 15) residues (Toma & Hatano 2007).

On the other hand, there are some studies which report N_2_O emissions of the same magnitude or even higher than those of the present study, e.g. Rizhiya *et al.* (2011) for wild cabbage leaves (C:N = 21, EFR 25 mg N_2_O-N/g residue-N in 50 days), Velthof *et al.* (2002) for white cabbage (C:N = 21), Brussels sprouts (C:N = 14), mustard (C:N = 10), or broccoli residues (C:N = 14) (EFR >35 mg N_2_O-N/g residue-N in 78 days) and Ruser *et al.* (2009) for mustard residues (EFR 31–37 mg N_2_O-N/g residue-N in a year). It is noticeable that all of these studies feature crop residues with C:N ratios <25 as was the case in the present study (C:N = 12). Increasing N_2_O losses at lower C:N ratios have been described before (Baggs *et al.* 2000; Delgado *et al.* 2010; Chen *et al.* 2013); however, presumably due to limiting factors other than N availability (e.g. C availability), this relationship can also be reversed, as reported by Frimpong & Baggs (2010; C:N = 6·8–11·7) and Huang *et al.* (2004; C:N = 8–118). The present results were obtained on a nutrient-poor sandy soil with a sole agricultural history and a fallow period of almost 2 years prior to the experiment. Hence, it can be assumed that readily available organic residues from previous crops had been absent, so that the capacity of the soil to deliver nutrients was relatively small. The situation on a soil continuously used for vegetable production may differ greatly. Therefore, differing crop histories should be considered carefully before transferring these results to other soil conditions. Finally, it should be noted that considerable uncertainties are associated with the estimated N_2_O emission sums in the present study due to the limited number of measurements at the time of the emission peak, which accounted for most of the emissions.

### Effect of residue placement on nitrous oxide emissions

Contrary to the proposed hypothesis, the cumulative emissions of N_2_O were of the same magnitude and in fact, although statistically insignificant, higher in the mulch than in the mix treatment. This implies that the accessibility of the residues to microorganisms was not limiting the N_2_O emission activity either because microorganisms quickly migrated into the mulch layer from the upper mineral soil or because the microbial community that was already on the crop residues was as capable of N turnover as the microbial community in the soil. The latter proposition is in line with findings by Flessa *et al.* (2002), who studied the decomposition and denitrification of grass mulch in soil compared to quartz sand and concluded that the ‘indigenous’ microflora on plant residues is the determining factor for decomposition and denitrification. It should be noted that in the present study the cumulative CO_2_ emissions during the early period after residue application, which represent microbial activity, were in fact lower in the mulch treatment compared with the mix treatment. Apparently this did not cause any limitation to N_2_O production. Shortly after application, the mulch became a layer of wet sludge covering the soil consistently, which probably featured favourable conditions for anaerobic fermentation processes. This assumption is supported by a strong putrid smell detectable for a few days at the time of peak emissions. The results suggest that surface-application of cauliflower leaf residues may produce as high or even higher N_2_O emissions than incorporation by homogeneous mixing with the topsoil. On the other hand, de Ruijter *et al.* (2010*b*) reported that surface application led to much lower denitrification losses (N_2_O+N_2_) than rototillage of leek, broccoli and sugar beet residues. Then again, the current findings are corroborated by results from Escobar *et al.* (2010) for soybean residues and from Baggs *et al.* (2003) for bean (*Vicia faba*) residues: those studies indicated higher N_2_O emissions from surface-applied compared with incorporated residues, which the authors attributed to a conservation of soil moisture and the concentration of O_2_ consumption as well as C and N availabilities in the upper mineral soil favouring the creation of microsites with high denitrification activity. In the present experiment, high variability in soil moisture was prevented by roofing/irrigation. This facilitated the comparison of treatments but natural variability might have produced different emission sums and courses, depending on the major location of emission sources. Whether the zones or microsites of high denitrification activity occur primarily in the mulch layer itself or in the upper millimetres of the mineral soil that receive infiltrate from the mulch layer or both remains to be investigated.

### Ammonia emissions

The measured maximum emission rates, emission sums, and EFRs for NH_3_ can be classified as low to medium considering the high N content (31 g N/kg DM) of the applied cauliflower residues. By comparison, de Ruijter *et al.* (2010*a*) indicated that NH_3_ emissions after surface-application of broccoli, leek, sugar beet, cut grass, fodder radish and mustard residues constituted between 50 and 160 mg NH_3_-N/g residue-N in 119 days while linearly increasing with residue N content (EFR of 60 mg NH_3_-N/g residue-N at 30 g N/kg DM). This magnitude of NH_3_ losses is consistent with results from Glasener & Palm (1995), who performed a laboratory incubation experiment and determined EFRs between 34 and 118 mg NH_3_-N/g residue-N for surface-applied residues of 10 tropical legume species in 3 weeks. Remarkably, the emissions can reach even higher levels, e.g. as described by Larsson *et al.* (1998) who found NH_3_ emission sums from surface-applied mulches ranging from 20 mg NH_3_-N/g residue-N (8 kg N/ha) for ‘low-N’-grass (12 g N/kg DM), over 170 mg NH_3_-N/g residue-N (170 kg N/ha) for alfalfa (43 g N/kg DM), to 390 mg NH_3_-N/g residue-N (190 kg N/ha) for ‘high-N’-grass (21 g N/kg DM) in 3 months. It seems unlikely that a significant part of the NH_3_ emissions in the present study was not covered by the experimental duration since NH_3_ losses appeared to have returned to background levels 1 month after application. Besides, the continuous ventilation of the chamber anchors in combination with the high uptake capacity of the filters probably created NH_3_ emissions that were unlimited in terms of air turbulence. This assumption is supported by the results of the pre-experiment, where a complete recovery of NH_3_ was found after 1-h filter exposition in the desiccator for simulated emission rates as high as 20 mg NH_3_-N/m^2^/h, compared with the maximum emission rates of 1·67 mg NH_3_-N/m^2^/h observed in the field. On the other hand, a substantial overestimation of NH_3_ emissions due to this technique is also unlikely since actual wind speeds in the field (0·4 m above surface) were >0·1 m/s for a proportion of 0·83 of the duration of the experiment. This translates to an air travelling distance of >15 chamber diameters/min, which is high in comparison with non-limiting flow-through-rates of >0·3 chamber volumes/min reported for emission rates of 5 mg NH_3_-N/m^2^/h (Janzen & McGinn 1991). However, in order to estimate absolute NH_3_ emission rates with greater certainty it is desirable to perform a calibration of this technique against well-established methods that determine absolute and actual *in situ* NH_3_ emissions under different weather conditions, as it has been done for a range of alternative methods (Pacholski *et al.* 2008).

### Effect of residue placement on ammonia emissions

Unlike the findings of several other studies and contrary to the hypothesis of the present study, the incorporation of crop residues did not reduce NH_3_ emissions as compared with surface-application of residues. In fact, neither the emission rates during the early period, i.e. at time of highest emissions, nor the total emission sums were significantly different between the two treatments. In contrast, results from de Ruijter *et al.* (2010*a, b*), Glasener & Palm (1995) and Janzen & McGinn (1991) suggested that NH_3_ emissions from crop residues are a surface phenomenon and disappear after incorporation of residue into the soil. Also, Mohr *et al.* (1998) indicated that NH_3_ emissions as high as 80 mg NH_3_-N/g residue-N from mulched alfalfa residues (37 g N/kg DM) in 95 days are reduced to 5 mg NH_3_-N/g residue-N when incorporated. To understand these differences one has to consider the processes of NH_3_ creation and volatilization: net production of NH_4_^+^, abiotic deprotonation of NH_4_^+^ (pH- and temperature-dependent), and transport of NH_3_ towards the atmosphere across the concentration gradient (diffusion- and wind-driven). First, the incorporation of residues may result in higher ammonification rates compared with surface application. However, incorporation may also produce higher rates of nitrification, immobilization and abiotic fixation of NH_4_^+^, so that the effect on net NH_4_^+^ production is very difficult to assess. Unfortunately, ammonification was not monitored in the present study. Secondly, it is known that decomposition of plant residues generally leads to an increase in pH value (Kimber 1973), which will facilitate the transformation of NH_4_^+^ to NH_3_, especially in the concentrated mulch layer. And thirdly, less NH_3_ derived from residues should reach the soil surface after incorporation compared with surface application due to the greater transport distance and associated passing of potential reaction sites. On the basis of this knowledge, the following possible explanations for the missing effect of residue placement on NH_3_ emissions are proposed. First, emissions were facilitated in the mix treatment by two factors: (a) the high diffusivity of this soil, in particular since plant residues may themselves have increased the porosity of the soil, and (b) enhanced net NH_4_^+^ production after incorporation into the soil as a result of increased accessibility of residues to microorganisms. The latter would fit in line with the higher CO_2_ emissions observed during the early period in the mix treatment. Secondly, emissions were hindered in the mulch treatment by the wet and compact mulch layer, which limited NH_3_ diffusion towards the surface.

## CONCLUSIONS

The presented N emission sums of 9·9 (mix) and 14·8 (mulch) kg (NH_3_-N+N_2_O-N)/ha in 65 days are negligible compared with N balance surpluses reported for 2-year crop rotations involving cauliflower (approx. 200 kg N/ha; Nett *et al.* 2011). Nevertheless, these emissions can be considered to be too high in view of their environmental impact. The annual losses of these gases may become even higher, especially when fertilization rates exceed recommended values and multiple ‘critical’ crops are cultivated per year. Moreover, some studies demonstrated that N_2_O emissions during the winter can be as high as those during the rest of the year (Kaiser & Ruser 2000). On the other hand, the application rate of residues used in the current experiment was exceptionally high, which may have produced particularly high emissions. The continuous irrigation in the current experiment probably produced favourable conditions for denitrification. However, irrigation could also have reduced NH_3_ emissions by enhancing the infiltration of NH_4_^+^ from the mulch layer into the soil and the leaching of soluble organic N compounds to deeper soil layers. The presented results for an irrigated sandy soil should not be transferred to other soil types and moisture conditions because residue decomposition and emissions of NH_3_ and N_2_O are known to be strongly influenced by soil texture and moisture. It is concluded that a high input of readily available organic C and N, such as with vegetable crop residues, can lead to substantial NH_3_ and N_2_O emissions even in coarse-textured soils where soil moisture remains far below the levels that are usually associated with high denitrification rates.

Special thanks go to Simone Starke and Ingo Hauschild for technical assistance. Financial support from the German Federal Agency for Agriculture and Food (BLE) is gratefully acknowledged.
